# Can The Moblees™ Move Canadian Children? Investigating the Impact of a Television Program on Children's Physical Activity

**DOI:** 10.3389/fpubh.2018.00206

**Published:** 2018-07-25

**Authors:** Guy Faulkner, Rebecca Bassett-Gunter, Lauren White, Tanya R. Berry, Mark S. Tremblay

**Affiliations:** ^1^School of Kinesiology, University of British Columbia, Vancouver, BC, Canada; ^2^School of Kinesiology and Health Science, York University, Toronto, ON, Canada; ^3^Faculty of Kinesiology and Physical Education, University of Toronto, Toronto, ON, Canada; ^4^Faculty of Kinesiology, Sport, and Recreation, University of Alberta, Edmonton, AB, Canada; ^5^Healthy Active Living and Obesity Research Group, Children's Hospital of Eastern Ontario Research Institute, Ottawa, ON, Canada

**Keywords:** children, physical activity, screen time, television, communication

## Abstract

**Background:** The effects of messaging about physical activity and sedentary behavior purposefully integrated into children's TV programming on children's behavior is unknown. The Moblees is a Canadian childrens' show that explicitly promotes physical activity. Two studies were conducted to (1) examine whether children were more physically active when watching a Moblees episode, and (2) explore parental perceptions of the show.

**Methods:** Study 1 was an experimental study with 21 families randomized to watch an episode of The Moblees vs. a control condition. Movement was assessed through accelerometry and observation. A Chi-square test was used to compare the direct observation proportions of children sitting between intervention and control conditions. Independent *t*-tests were performed to examine the differences in total vector magnitude counts between the experimental and control groups. Study 2 was an online cross-sectional study with 104 parent/child dyads that included viewing an episode of The Moblees. To identify correlates and predictors of parent-reported child PA during viewing The Moblees compared to other TV programs, Pearson's correlations and a linear regression were calculated, respectively.

**Results:** In study 1 there was a significant association between condition and whether or not children remained sitting χ^2^ = 55.96, *p* < 0.001. There was a significant difference in counts between the two conditions, *t*_(13, 61)_ = 2.29, *p* < 0.05. Children randomized to the experimental group (i.e., Moblees) moved more compared to control. In study 2 the majority (76%) of parents reported that their child engaged in some physical activity during viewing. Parent encouragement during viewing was the strongest predictor of child physical activity while viewing (β = 0.30, *p* < 0.01).

**Conclusion:** Television content that includes messaging about physical activity and sedentary behavior, and positive portrayals of physical activity may influence the physical activity of young children. Although the benefits of such modest movement are not clear without further evidence of accumulation over time and/or transfer to other settings, television programming might provide a far reaching medium for knowledge translation.

## Background

Screen-based sedentary behavior (SSB) and physical inactivity are highly prevalent and increasing among Canadian children. On average, Canadian children aged 3–5 years spend about half of their waking hours in sedentary pursuits and an average of 2 h per day in front of screens (1). Only 15% of Canadian children aged 3–4 years meet the Canadian Sedentary Behavior Guidelines for the Early Years, which recommend that daily screen time (i.e., use of computers, television, etc.) be limited to <1 h (2). Effectively tackling rising rates of physical inactivity and SSB among Canadian children will require population level intervention (3).

Strategies to reduce SSB and increase physical activity among children should involve both parents and children to foster behavior change (4). Appropriate targeted messaging about physical activity and SSB should be one component of such interventions. How to create and disseminate persuasive messages at a national level remains a challenge. Could a children's television (TV) show be one (perhaps counterintuitive) answer? A recent media content analysis of 82.5 h of children-specific TV broadcasting on UK and Irish channels found that physical activities were commonly integrated into TV programming, and health and physical activity promoting segments were prominent and portrayed positively (5). Intervening to deliver physical activity messages within the context of what many children are already doing, watching screens, is a novel and likely necessary development to promote healthy active living. Yet, the effects of physical activity and SSB messages purposefully integrated into children's TV programming on children's behavior is unknown.

### The moblees™

The Moblees is a live-action mini-musical adventure series currently airing daily, nationally on the Canadian Broadcasting Corporation (CBC) during their children's TV block, CBC Kids. It was launched December 22nd, 2014. The Moblees embraces an anti-sedentary philosophy seeking to create a “movement movement” among young children and their families in Canada (aged 3–6 years). The Moblees takes a different approach to increasing children's physical activity. Rather than aiming to reduce SSB *per se*, The Moblees seek to increase physical activity and decrease sedentary behaviors among children *while* they are watching an informative TV program, while also instilling the importance of movement throughout the day.

The first season of The Moblees TV program included a series of episodes containing compelling characters, catchy music, and movement-centric journeys. Each episode contains the characters of Bailey Butterfly, Carlin Caterpillar, Gisbert Grasshopper, Dasha Dog, Sylvio Snake, and YOU [the watching child]—to complete creative journeys. Within each episode, the characters encounter various obstacles and problems that can only be solved with movement. For example, in the “Purple Party Twist” episode (see https://www.youtube.com/watch?v=MU0NW-wRpUM — geofenced within Canada; other examples can be searched for on YouTube), the purple grape juicer breaks down. In order for everyone to have grape juice for the party, each character and YOU must smash the grapes down and create juice by engaging in the “twist” movement. The characters invite each watching child (the “home-doer”) to join in on the twist movement to make more juice available for everyone attending the party. Each episode integrates non-sedentary behaviors and encourages children's active movements throughout, with messaging to continue active movement for the rest of the day. All episodes follow a similar narrative arc leading to an active component approximately every 3 min. To complement the show, there are live-stage concerts, mobile applications, digital content, an education outreach program, and the online Moblees Medal Program (www.TheMoblees.com). Overall, the stated intent of The Moblees is to “address the obesity epidemic by fostering non-sedentary, physically active behaviors, and educating and instilling healthy nutritional habits in children.”

There is of course a tremendous paradox in using a TV show to promote physical activity and messages about reduced SSB. Yet, screens are not going to go away. In the same fashion that “exergaming” may be used to offset SSB while engaging in video games (6), it is possible that targeted programming and messaging could be used to decrease SSB during TV viewing. Further, it is possible that such programming and messaging could promote physical activity more broadly while also targeting parents' attitudes and support behaviors regarding physical activity and reduced SSB. Parents' attitudes and beliefs about physical activity and SSB are related to their support behaviors as well as their children's physical activity and SSB behavior (4, 7). Physical activity messages targeting parents through TV commercials may be effective in changing parents' support behaviors for children's physical activity (8). However, physical activity promotion within a TV context more broadly and its impact on subsequent childhood behavior remains little examined (5). In Canada, The Moblees' approach is attempting to modify the content of what children are watching in ways that increase physical activity and reduce sedentary behavior the rest of the day. To our knowledge, such an approach has not been evaluated.

Given the national reach of The Moblees, this represented a novel, “natural” intervention (9) with potential for high impact in communicating healthy active living messages to young children and their parents at a population level. Accordingly, two studies were conducted: Study 1 examined whether children were more physically active when watching a Moblees episode vs. a control TV program; Study 2 examined (a) parent-reported child PA while viewing an episode of The Moblees, (b) parent perceptions of the program, and (c) relationships between parents' perceptions of the program, parents' support behavior for physical activity and reduced SSB, and children's physical activity during the show.

## Study 1

The purpose of this study was to examine children's physical activity while viewing The Moblees compared to a control TV program. It was hypothesized that children would be more physically active while watching The Moblees compared to the control condition.

### Method

#### Participants

Families with children between the ages of 3 and 6 years were purposively recruited to participate in this study. Twenty-one families with 25 children between the ages of 3 and 6 years participated in the study. Eleven families (*n* = 12 children) were randomized into the experimental group (i.e., viewed the first episode of The Moblees—“Purple Part Twist”) and 10 families (*n* = 13 children) into the control condition (i.e., viewed a program called You & Me). You & Me is a children's TV show that has no explicit promotion of movement but encourages the child to interact with the show characters (see https://www.youtube.com/watch?v=Cy0jszYDqWQ&list=PLPZ40gM7aDFCJnEj1qkNLRQHTCWb0hJvP&index=33). The majority (76%) of parents were mothers between the ages of 34 and 49 years and were highly educated (90% with university or postgraduate education). The mean age in the experimental condition was 40.45 years and 39.3 years in the control condition. Children included both girls (48%) and boys (52%) with a mean age of 5 years. There was an equal number of mothers in each group and also individuals with an undergraduate education or greater. There were no significant differences between the two groups on any demographic variables.

#### Procedure

Families were told the study was assessing perceptions of two new CBC shows with no further explicit information provided about the nature of the study. After obtaining informed consent, two research assistants attended a study session at each participant's house. Ethics approval was obtained from the University of Toronto Research Ethics Board and informed consent was obtained. Each parent-child dyad was randomized to watch one of two TV shows. Randomization was performed using an allocation ratio (1:1) to experimental or control groups. This occurred by using the coin toss function on an online random number generator (i.e., random.org). Participants randomized to the experimental group watched an episode of The Moblees. The control group watched an episode of You & Me. Both episodes were 11-min in length, included live-action characters, and were broadcast on the CBC Kids TV channel for children aged 6 years and under.

After randomization, at the beginning of the study session, parents completed a short demographic questionnaire. Parents and their child then viewed the 11-min episode together. During this time, children wore an ActiGraph GT3X+ (ActiGraph, Pensacola, FL) accelerometer on their right hip. The ActiGraph GT3X+ is a tri-axial accelerometer that was used to measure movement accelerations in counts per minute during each episode viewed. Epoch time was set to 1 s. Parents in both conditions were instructed to encourage their children to follow along with the prompts from the episode. A modified version of the System for Observing Fitness Instruction Time [SOFIT; (10)] was also used to record movements during the entire 11-min episode. At 30-s intervals, children were recorded as either lying, sitting, standing, walking, vigorous, or other. Children's movements were classified as follows: (a) vigorous when they were leaping, jumping, squatting, or flapping arms, or (b) other when they were pushing a button or leaning side to side while remaining seated. Parents received a $25.00 gift card for participation.

### Data analysis

Descriptive analyses were performed for all variables. For the purpose of this study, only the total number of children sitting every 30 s, in each condition, is presented. A Chi-square test was used to compare the direct observation proportions of children sitting between intervention and control conditions. To gauge relative activity, total vector magnitude counts for the 11-min bout were extracted using Actilife software (ActiGraph, Pensacola, FL). Independent *t*-tests were performed to examine the differences in counts between the experimental and control groups. Significance was assessed at α = 0.05 and analyses were conducted using SPSS Statistics (version 23).

### Results

#### SOFIT data

**Figure 1:**
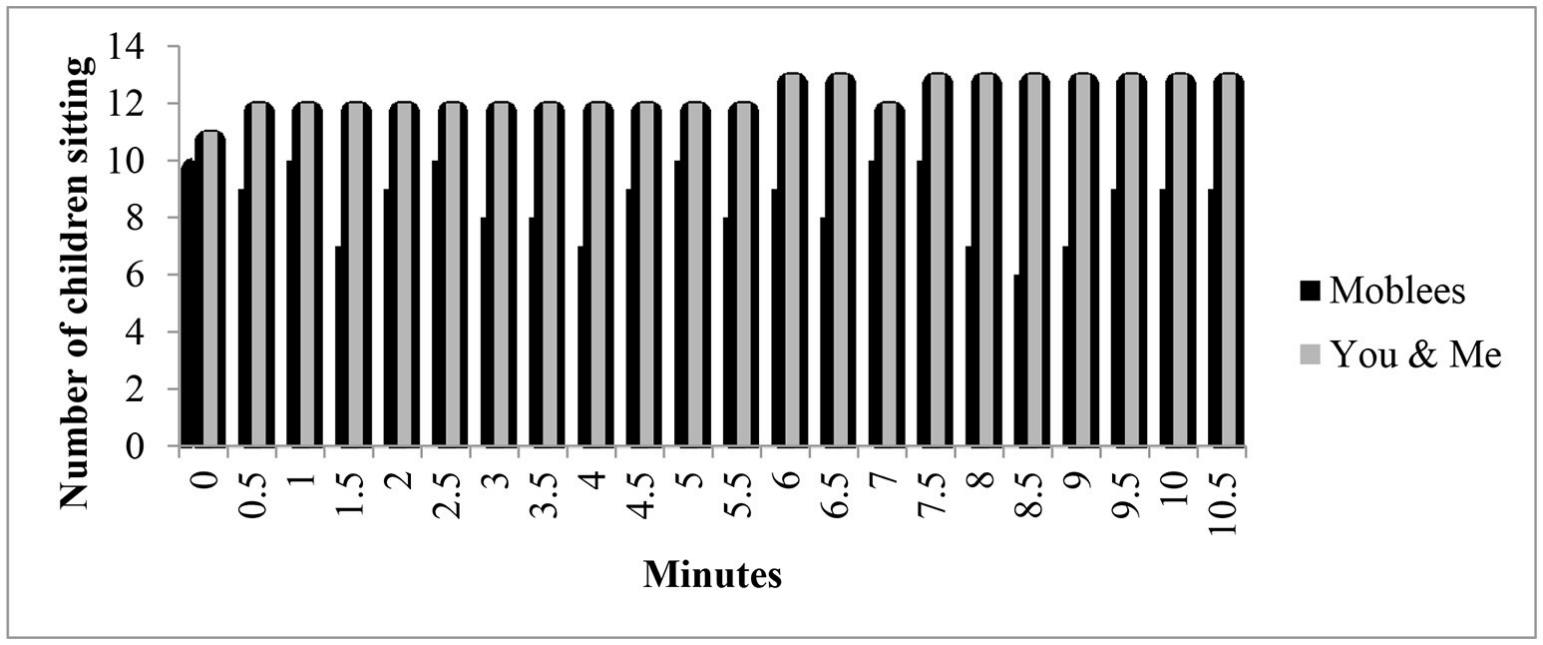
Number of children sitting during each episode reported by SOFIT 30-s intervals.

Children randomized in the control condition mostly sat throughout the entire show compared to children in the experimental group (see Figure 1). Considering all observation points, sitting was observed in 95% of cases during the control condition and 72% of cases during the Moblees condition. This was a significant difference (χ^2^ = 55.96, *p* < 0.001).

#### Accelerometry data

**Figure 2:**
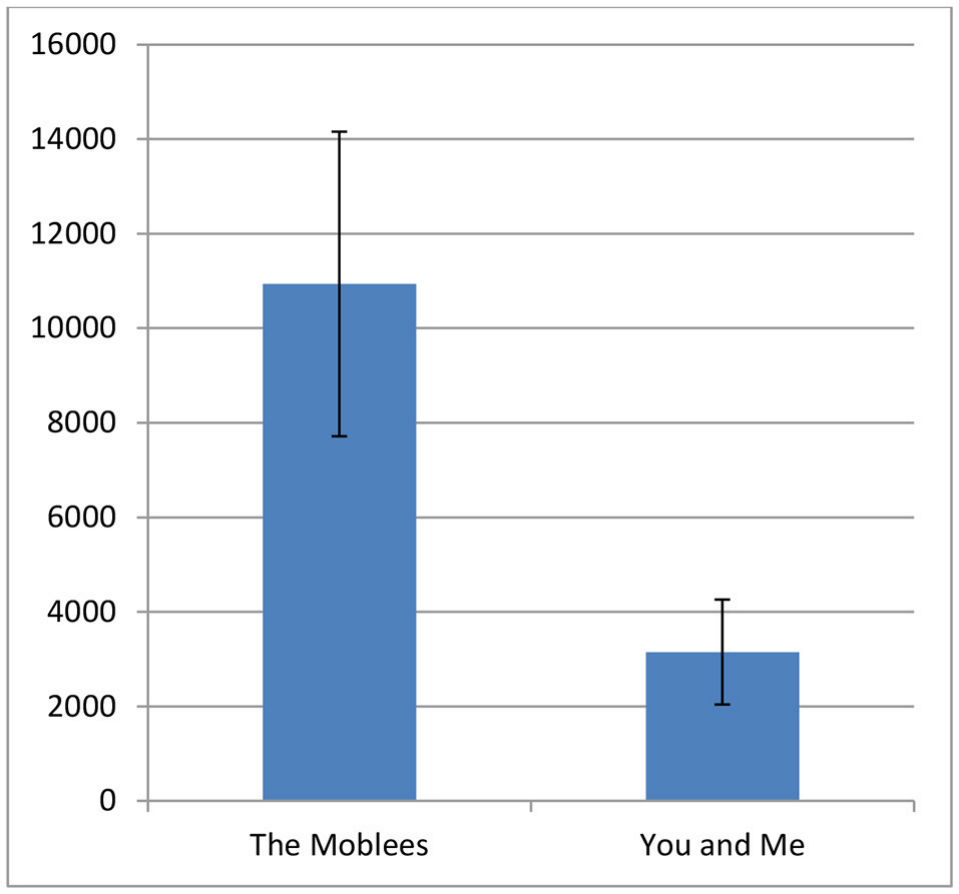
Average vector magnitude counts with standard errors for each condition. There was a significant difference in counts between the two conditions, *t*_(13, 61)_ = 2.29, *p* < 0.05.

There was a significant difference in counts between the two conditions, *t*_(13, 61)_ = 2.29, *p* < 0.05. Those children who were randomized to the experimental group (i.e., Moblees) moved more compared to control (i.e., You & Me; see Figure 2).

## Study 2

The purposes of this exploratory study were to (a) examine parent-reported child physical activity while viewing The Moblees, (b) examine parent perceptions of the program, and (c) identify relationships between parents' perceptions of the program, parental support behaviors for physical activity and reducing SSB, and children's physical activity during the show.

### Method

#### Participants

Participants included parents of children between the ages of 3–6 years (*N* = 104; *M*_age_ = 34.7 years) recruited through online classifieds, social media, community postings, and word of mouth. Participants were mostly married (91%) mothers (90%) with some post-secondary education (97%). The child participants (i.e., child who viewed The Moblees during the study) included girls (54%) and boys (46%) who were on average 4 years old.

#### Procedure

Participants completed an online questionnaire assessing (a) demographic variables, (b) perceptions and experiences regarding TV viewing and SSB, and (c) attitudes and intentions regarding support for child physical activity and reduced SSB. After completing the baseline questionnaire, parents viewed an episode of The Moblees with their child. Immediately after viewing the episode, parents completed a thought listing exercise (11) to document their perceptions of the program. Parents also completed a follow-up questionnaire assessing (a) parental self-report of child's PA behavior during the program and (b) encouragement provided during viewing. Approximately 6 months after the initial participation, parents were invited to complete an additional questionnaire assessing their child's subsequent engagement with The Moblees program. Approximately 55% of the original sample completed the 6-month follow-up survey (*n* = 57). Parents received a $25.00 gift card for their initial participation, and a $5.00 gift card for their participation in the follow-up questionnaire. All procedures were approved by the institution's Research Ethics Board and informed consent was obtained.

#### Baseline measures

##### Parent behavior

Parents' self-reported physical activity over the past 7 days was assessed using the International Physical Activity Questionnaire Short Form (12). Parents' TV viewing over the past 7 days was self-reported in hours/minutes per day.

##### TV viewing and SSB experiences and perceptions

Parents reported on their rules or policies around TV viewing/SSB, how frequently they ate meals in front of the TV, their beliefs regarding the role of TV in providing educational and/or entertaining content for children, and how frequently they use the TV to entertain their child while they do other things. These items were adapted from He et al. (4) and were assessed on five-point likert-type scales.

##### Attitudes and intentions regarding child physical activity and SSB

Parents' attitudes and intentions toward (a) SSB reduction and (b) support for child physical activity were measured with four items developed based on recommendations for assessing these constructs (13). For example: I intend to help my child meet the screen time behavior guidelines (defined as <1 h per day for children ages 3–4 and <2 h per day for children ages 5–6) over the next week by limiting the amount of time my child can watch TV (or shows on an iPad) in a day (one *strongly disagree* to five *strongly agree*).

#### Post viewing measures

##### Child physical activity during viewing the moblees

Two items were used to assess parent reports of physical activity during Moblees viewing: (1) When your child was being asked questions by the characters, did he/she respond? If yes; Verbally (i.e., spoke to the characters but were not physically active); Physically (i.e., repeated the actions); Both (i.e., verbally and physically). This item was coded into a binary variable whereby children who did not respond or responded verbally were coded as “no PA” and children who responded physically or both verbally and physically were coded as “some PA.” (2) Overall, did you get the sense that your child was more physically active watching The Moblees than during other TV shows? Parents answered on a five-point scale (one *was not more physically active* to five *was a lot more physically active*).

##### Parent encouragement during viewing

One self-report item was used to measure parent encouragement during viewing: Did you encourage your child to respond? Parents answered on a five-point scale (one *never encouraged* to five *always encouraged*).

##### Parent perceptions of the moblees

Parents engaged in a thought listing exercise (11) to document their perceptions of the program. Parents were asked to record any thoughts that they had while viewing the program.

#### Six month follow-up

##### Subsequent engagement with the moblees

Participants reported whether or not their child had engaged in various aspects of The Moblees program since the initial study. Participants reported Yes or No to indicate their child's engagement in the following: watched The Moblees (online, CBC broadcast, or both), observed promotions, attended concerts, enrolled in medals program, child recalled pledge, parent recalled pledge, used The Moblees as a tool for child physical activity, parent encouraged child to watch The Moblees.

### Data analysis

Data were cleaned and screened for violations of statistical assumptions (14). Descriptive statistics were calculated to examine child PA during viewing. ANOVA was used to examine group differences between children who reportedly engaged in PA during viewing compared to children who did not (binary variable). To identify correlates and predictors of parent-reported PA during viewing The Moblees compared to other TV programs (continuous variable), Pearson's correlations and a linear regression were calculated, respectively. Thought listing data were analyzed via content analysis whereby parents' thoughts were coded as favorable, unfavorable, neutral or irrelevant [see (11)] by two coders. Of the original 406 thoughts (coding units) there were only 20 discrepancies between the two coders. For each of these, the thought was discussed between both coders and resolved to the most appropriate category. Finally, descriptive statistics were calculated to describe the 6-month follow up data.

### Results

#### Children's physical activity during viewing

The majority (76%) of parents reported that their child engaged in some physical activity during viewing (binary variable coded “some” vs. “none”). When asked if their children were more physically active during The Moblees compared to other TV shows, parents reported that their children were somewhat more active (*x* = 3.30 ± 1.52). Compared to those who did not, children who did engage in physical activity while viewing watched less TV overall (*F* = 3.9, *p* = 0.05) and received more parental encouragement for physical activity during the show (*F* = 5.5, *p* = 0.02). The following variables were correlated with child physical activity during the program (see Table 1): frequency of watching TV during meals (*r* = 0.36, *p* < 0.01), parents' intentions to support physical activity (*r* = 0.23, *p* < 0.05), parents' attitudes regarding reduced SSB (*r* = 0.27, *p* < 0.01), and parents' encouragement for physical activity while viewing The Moblees (*r* = 0.40, *p* < 0.01). All significant correlates were included in the regression model predicting children's physical activity during viewing (continuous variable). The model explained 23% of the variance in child physical activity while viewing (see Table 2). Parent encouragement during viewing was the strongest predictor of child physical activity while viewing (β = 0.30, *p* < 0.01).

**Table 1:** Descriptive data and correlations.

	**1**	**2**	**3**	**4**	**5**	**6**	**7**	**8**	**9**	**10**	**11**	**12**	**13**
PA During Moblees	3.3 (1.52)	0.15	0.18	0.13	0.36^**^	0.03	0.1	−0.04	0.03	0.23^*^	0.27^**^	0.07	0.40^**^
TV Rules		3.42 (0.92)	0.01	−0.16	0.14	0.30^**^	–0.27^**^	−0.03	0.32^**^	0.27^**^	0.48^**^	0.56^**^	0.10
Parent PA			3.51 (1.1)	0.05	0.09	−0.03	0.05	−0.21^*^	0.05	0.31^**^	0.16	0.09	0.04
Parent TV				3.46 (0.92)	0.32^**^	−0.05	−0.09	0.26^**^	0.04	0.10	0.05	−0.07	0.07
TV Meals					2.33 (1.34)	0.01	0.09	0.23^*^	−0.12	0.14	0.17	0.12	0.25^*^
TV Education						4.39 (0.92)	−0.14	0.09	0.33^**^	0.16	0.27^**^	0.36^**^	0.12
TV Entertain							2.62 (1.1)	−0.11	−0.20^*^	−0.26^**^	−0.23^*^	−0.34^**^	−0.14
TV Distract								3.53 (0.96)	0.12	0.08	−0.003	0.09	0.25
ATT-PA									4.07 (0.79)	0.62^**^	0.44^**^	0.49^**^	0.03
INT-SS-PA										4.15 (0.69)	0.57^**^	0.48^**^	0.23^*^
ATT SSB REDU											4.07 (0.75)	0.65^**^	0.17
INT-SSB-REDU												4.05 (0.79)	0.16
ENCOURGE-PA													3.73 (1.23)

Sample means (standard deviations) are shown on the diagonal.

** Correlation is significant p < 0.01.

* *Correlation is significant p < 0.05. TV during meals, TV MEALS; Parent attitude toward PA, ATT-PA; Parent intent to provide social support for PA, INT-SS-PA; Parent attitude toward SSB reduction, ATT-SSB; Parent intention toward SSB reduction, INT-SSB-RED; Parent encouragement for PA during the show, ENCOURGE-PA*.

**Table 2:** Predictors of child physical activity while viewing The Moblees.

	***R***	***R*^2^**	**Adjusted *R*^2^**	***B***	**Sig**.
	0.507	0.258	0.227		
Encourage PA				0.297	0.002^**^
Att-SSB				0.133	0.223
TV meal				0.259	0.006^**^
Int-SS-PA				0.047	0.665

Dependent variable: Child PA while viewing The Moblees. All significant correlates of PA were included in the model. Predictors: ENCOURG-PA, ATT-SSB, TV MEAL, and INT-SS-PA.

** *p < 0.01*.

#### Parent reactions to the moblees program; thought listing exercise

Participants listed a total of 406 thoughts regarding the show (approximately 4 thoughts per participant). The large majority (334; 82%) of parents' thoughts were favorable (e.g., “The show was fun for my child, she took an interest in it”), while the minority (43; 11%) were not favorable (e.g., “My son wandered away twice during the video as it didn't hold his attention”). Some parents listed neutral (11; 3%) (e.g., “I wonder if my daughter would have danced if she wasn't tired”), and irrelevant thoughts (18; 4%). Almost all of the parents (*n* = 97; 93%) said that they would let their child watch The Moblees in the future.

#### Six-month follow-up: engagement with the moblees program

Parent and child engagement with The Moblees program varied depending on the type of engagement. Overall, 39% of the sample reported watching the television program again since the initial study participation. Frequency of viewing the program ranged from one to six episodes (*x* = 3 episodes). Most children who watched the program did so on broadcast television (59%) or online (32%). Less than 20% of the sample engaged in other aspects of The Moblees program such as attending concerts, enrolling in the medals program or recalling the pledge. Some parents (19%) did use The Moblees to encourage physical activity among their children. See Table 3 for complete details regarding engagement.

**Table 3:** Engagement with the moblees at 6-month follow-up (*N* = 57).

**Engagement variable**	**Participant engagement**
	***N*** **(%)**
Watch the Moblees	22 (39%)
Online	7 (32%)
CBC	13 (59%)
Both online and CBC	2 (9%)
Observe promotions	12 (21%)
Attend concert tour	8 (14%)
Enroll in medals program	6 (10%)
Child recall pledge	7 (12%)
Parent recall pledge	7 (12%)
Parent use the Moblees as tool for child PA	11 (19%)
Parent encourage child to watch the Moblees	8 (14%)

## Discussion

Television content that includes messaging regarding healthy active living, and positive portrayals of physical activity could influence the physical activity and SSB of young children; appropriate television programming has been suggested as one strategy to address the epidemic of pediatric inactivity and obesity (5). Our findings provide some preliminary support for such a claim. The first study provides proof of principle in a TV show having an acute effect on physical activity and SSB of young children. Children viewing The Moblees episode moved more and sat less than those watching an interactive show that did not explicitly include messaging about physical activity. While the health benefits of this particular physical activity alone are likely negligible, the results suggest there is value in further research to determine whether children become habituated to viewing episodes and respond similarly over time. Additional research is also necessary to determine whether there is transferability beyond the viewing context such that physical activity and SSB are impacted throughout the day. For example, do children mimic the characters or activities on the playground? Do children learn about the benefits of physical activity and reducing screen time? While there is evidence that educational television programs can broaden young children's knowledge in general (15), assessment of these critical outcomes was beyond the scope of this preliminary study.

Although ascertained through self-report, most Study 2 parents also reported that their child was active watching the episode. Correlates of children's physical activity during viewing included parents' attitudes regarding SSB reduction and parents' intentions to support their child's physical activity. There was also an association between eating meals in front of the TV and being active during the episode. Speculatively, it may be possible that children who eat meals in front of the TV watch more TV in general (4) and as a result have prior experience of engaging with program content. Notably, the strongest predictor of physical activity during viewing was parental encouragement during the show. In study 1, all parents were instructed to encourage their child to interact with the episode (i.e., encourage children to respond to characters). It is possible that many young children may not independently engage in physical activity during viewing and require encouragement from a parent. It has been recommended that interventions to decrease SSB among children engage both parents and children (4). Recent recommendations regarding app use among preschoolers also include the need for design for a dual audience (i.e., both parent and child) to facilitate family participation in media use (16). Collectively, this literature and our findings suggest parental engagement with the show is an important precursor of behavioral engagement by the child. Alternatively, such parental encouragement may be less critical once a child becomes familiar with a show and its characters.

Given the positive reception to the episode by parents and children, The Moblees might be one effective medium for knowledge exchange. For example, Canada recently released the first 24-h Movement Guidelines for Children and Youth which integrate recommendations regarding physical activity, sedentary behavior, and sleep (17). Integrating messaging about the guidelines within The Moblees may be one cost-effective strategy to increase awareness of the guidelines among parents and other stakeholders (18). Future opportunities for knowledge exchange should be explored and evaluated while considering the low levels of parent and child engagement with The Moblees program 6 months following the initial viewing. Additional efforts to foster parental awareness and engagement may be needed to optimize the reach of the program for such purposes. This may not be easy in a context of many alternative programs, channels (regular public broadcasting vs. “on demand” streaming), media (e.g., apps, games) and platforms (e.g., television, ipad, phone). Future research may focus on understanding optimal strategies for engaging the target audience.

There are a number of limitations to the two studies. First, the sample of parents was mostly highly educated and of medium to high socio-economic status. Data from the US indicate that low-income families were most likely to be “media-centric” families whose households were saturated with background media throughout the day (19). Children from higher-educated, upper-income families spend less time with media than other children do (20). Children and parents from more diverse backgrounds may have experienced The Moblees quite differently. Second, study two was conducted online and relied on self-report measures where responses may be subject to social desirability bias. Third, the television program chosen for the control condition for study one should be considered; the show encouraged child interaction but did not explicitly encourage movement. It was also broadcast by the same company and was similar in length. A more active contrasting condition could have resulted in different findings. Finally, parents in study one were told to encourage their child to interact with the show when guided by the television characters. This may not reflect actual practice in the home. All parents were also told the purpose of the study was to assess two children's television shows with no mention in advance of The Moblees. Despite randomization there may have been some parental reactivity based on condition that influenced their encouragement of their child.

Caution is required in drawing conclusions. Given the potential for national reach, the program may hold promise as a tool for communicating physical activity and sedentary behavior messages to Canadian children and parents. An episode of The Moblees may be effective for encouraging movement during viewing among young children although the benefits of such modest movement are not clear without further evidence of accumulation over time and/or transfer to other settings. This preliminary research contributes to very limited evidence concerning the use of childrens' TV programming for promoting physical activity messaging (5). Further research, perhaps using media use diaries completed by parents, is needed to identify whether there are any sustainable benefits for children and parents.

## Ethics statement

The protocol was approved by the Research Ethics Board from the University of Toronto and York University. All subjects gave written informed consent in accordance with the Declaration of Helsinki.

## Author contributions

All authors contributed to the conception, design and interpretation of the studies. GF and LW were responsible for the acquisition of data and analysis for study 1. RB-G was responsible for the acquisition of data and analysis for study 2. GF drafted the article and all authors contributed to revising it critically for important intellectual content. All authors gave final approval of the final draft.

### Conflict of interest statement

GF, RB-G, and MT received consulting fees from the developers of The Moblees during the early development of the concept. The remaining authors declare that the research was conducted in the absence of any commercial or financial relationships that could be construed as a potential conflict of interest.
